# Influence of Galectin-9 Treatment on the Phenotype and Function of NK-92MI Cells in the Presence of Different Serum Supplements

**DOI:** 10.3390/biom11081066

**Published:** 2021-07-22

**Authors:** Matyas Meggyes, David U Nagy, Timea Balassa, Krisztina Godony, Agnes Peterfalvi, Laszlo Szereday, Beata Polgar

**Affiliations:** 1 Department of Medical Microbiology and Immunology, Medical School, University of Pecs, 12 Szigeti Street, 7624 Pecs, Hungary; balassa.timea@pte.hu (T.B.); szereday.laszlo@pte.hu (L.S.); polgar.beata@pte.hu (B.P.); 2 Janos Szentagothai Research Centre, University of Pecs, 20 Ifjusag Street, 7624 Pecs, Hungary; 3 Medical Centre, Cochrane Hungary, University of Pecs, 7623 Pecs, Hungary; davenagy9@gmail.com; 4 Department of Obstetrics and Gynaecology, Medical School, University of Pecs, 17 Edesanyak Street, 7624 Pecs, Hungary; godony.krisztina@pte.hu; 5 Department of Laboratory Medicine, Medical School, University of Pecs, 13 Ifjusag Street, 7624 Pecs, Hungary; agnes.peterfalvi@gmail.com

**Keywords:** NK-92MI, Galectin-9, TIM-3, cytotoxicity

## Abstract

Galectins are one of the critical players in the tumor microenvironment–tumor crosstalk and the regulation of local immunity. Galectin-9 has been in the limelight in tumor immunology. Galectin-9 possesses its multiplex biological functions both extracellularly and intracellularly, plays a pivotal role in the modulation of adaptive and innate immunity, and induces immune tolerance. NK-92MI cell lines against different malignancies were extensively studied, and recently published trials used genetically chimeric antigen receptor-transfected NK-92MI cells in tumor immunotherapy. Besides the intensive research in tumor immunotherapy, limited information is available on their immune-checkpoint expression and the impact of checkpoint ligands on their effector functions. To uncover the therapeutic potential of modulating Galectin-9-related immunological pathways in NK-cell-based therapy, we investigated the dose-dependent effect of soluble Galectin-9 on the TIM-3 checkpoint receptor and NKG2D, CD69, FasL, and perforin expression of NK-92MI cells. We also examined how their cytotoxicity and cytokine production was altered after Gal-9 treatment and in the presence of different serum supplements using flow cytometric analysis. Our study provides evidence that the Galectin-9/TIM-3 pathway plays an important role in the regulation of NK cell function, and about the modulatory role of Galectin-9 on the cytotoxicity and cytokine production of NK-92MI cells in the presence of different serum supplements. We hope that our results will aid the development of novel NK-cell-based strategies that target Galectin-9/TIM-3 checkpoint in tumors resistant to T-cell-based immunotherapy.

## 1. Introduction

According to data from the World Health Organization (WHO) and the latest update from the International Agency for Research on Cancer (IARC) [1], cancer is the second leading cause of death globally, with an estimated 18.1 million cases and 9.6 million cancer deaths. This number is predicted to increase up to 29.4 million cases by 2040.

In the past decades, the therapeutic arsenal against malignant tumors has been mainly composed of conventional forms of therapy such as surgery, radiation, or chemotherapy. In recent years, tumor immunotherapy has become a novel, promising therapeutic alternative for cancers. As this treatment regimen is highly effective, less toxic, more tolerable, and provides numerous benefits, it has now become one of the first choices against many malignant neoplasms [2].

Natural killer (NK) cells are an essential part of the innate immune system with both effector and immunoregulatory functions. The cytotoxic activity of the NK cells can be triggered without prior sensitization or immunization in a major histocompatibility complex (MHC)-unrestricted manner and [3] firmly regulated by various activating and inhibitory receptors, depending on the presence of their ligands on the target cells and the activation state of the NK cells. Furthermore, NK cells are also capable of promoting inflammation and regulating Th1-type immunity by releasing pro-inflammatory or regulatory cytokines, chemokines, and RANTES [4], or by interacting with antigen-presenting sentinel cells such as macrophages, dendritic cells, and pathogen-infected host cells [5]. These large granular lymphocytes play a pivotal role in the defense against virally infected and malignant cells; therefore, they are suitable for adoptive immunotherapy [6,7]. For tumor therapy, adoptive transfer of expanded and in vitro-activated NK cells has been widely used [7]. In clinical trials, sources of the NK cells could be from autologous patients [8], allogeneic donors [9], cord blood-derived NK cells [10], induced pluripotent and human embryonic stem cells (iPSCs and hESCs), or NK cell lines [11]. The latter source offers several benefits as cell lines are free of contaminating T or B cells, thus mitigating any alloreactive or graft-versus-host disease (GvHD) effects that may be associated with blood-derived NK cells.

The NK-92 cell line (ATCC^®^ CRL-2407TM) was originally established in 1994 from the peripheral blood mononuclear cells (PBMC) of a 50-year-old Caucasian male patient with rapidly progressive non-Hodgkin’s lymphoma, whose marrow was diffusely infiltrated with large granular lymphocytes [12]. Later, an interleukin (IL)-2 independent variant—the NK-92MI cell line (ATCC^®^ CRL-2408TM)—was derived from the NK-92 cells by stably transfecting the parental cells with human IL-2 cDNA in the retroviral MFG-hIL-2 vector using a particle-mediated gene transfer technology. Immunophenotypic analysis of NK-92 and NK-92MI cell lines revealed that they display the characteristics of activated NK cells and express common surface markers, such as CD2, CD7, CD11a, CD28, CD45, CD54 (ICAM-1), and CD56. However, they showed negative labeling for CD1, CD3, CD4, CD5, CD8, CD10, CD14, CD16, CD19, CD20, CD23, CD34, and HLA-DR molecules [13,14,15]. In contrast to primary NK cells, these cell lines do not bear CD16, lack NKp44 and NKp46 killer activation receptors (KAR), and do not express inhibitory killer cell immunoglobulin-like receptors (KIR) except for KIR2DL4 [16]. However, it should be noted that DNA methylation studies have suggested that NK-92 cells possess various KIRs [12]. The absence of most of the KIRs in NK-92 and NK-92MI cell lines results in higher cytotoxic response and lytic activity than primary NK cells to human malignant cells. In functional studies, NK-92 cells showed a strong anti-leukemia effect both in vitro and in vivo, making them an ideal subject for adoptive tumor therapy [16,17]. Additionally, NK-92MI cells also showed high cytotoxic activity against different cancer cell lines in combination with monoclonal therapeutic antibodies [18]. Following preclinical development [19], a completed phase I dose-escalation trial in advanced cancer patients with renal cell cancer and melanoma established the feasibility and safety of the large-scale expansion and administration of NK-92 cells in allogeneic cellular tumor immunotherapy [11]. Finally, these highly cytotoxic cell lines are still the most widely examined and used models for evaluating NK-mediated cytotoxic activity in in vitro functional assays [13].

It has become scientifically proven that the tumor microenvironment (TME) critically suppresses the execution of tumor-specific immunity, resulting in immune escape of malignantly transformed cells. In fact, recently, it was found that the glycan-binding galectins are one of the critical players involved in the TME–tumor crosstalk and in the regulation of local immunity. Among the 15 known human galectins, Galectin-1, -3, and -9 (Gal-9) have been in the limelight in tumor biology.

A member of the galectin family, the tandem-repeat Galectin-9 (Gal-9) is a β-galactoside-binding 36 kDa protein with a conserved carbohydrate recognition domain [20]. Although it was first described as an eosinophil chemoattractant, its role in the immunological context was extensively studied and clearly verified by numerous research groups and papers during the past decade [21,22,23]. Gal-9 is expressed ubiquitously in normal tissues and cells with particular dominance in the epithelial, endothelial, and immune cells. Furthermore, its expression has also been detected during pathological conditions in various hematological malignancies and solid tumors [24,25].

Emerging evidence indicates that Gal-9 possesses its multiplex biological function both extracellularly and intracellularly. The secreted, extracellular Gal-9 plays a pivotal role in the modulation of adaptive and innate immunity, regulates mucosal immunity, and induces immune tolerance. As a pleiotropic regulator, it suppresses the Th1- and Th17-type immune response and activates regulatory T cells (Treg) both in vitro and in vivo [26,27], which explains its anti-inflammatory role in animal models of autoimmune and allergic disorders (e.g., neuroinflammation, experimental autoimmune encephalomyelitis, and autoimmune arthritis) [28,29,30]. On the contrary, other papers reported that Gal-9 might promote Th1-type immunity under certain physiologic or pathologic conditions, such as during activation of mononuclear phagocytes and dendritic cells, in anti-bacterial immunity allograft rejection, joint inflammation, skin allergy, or endometriosis [31,32,33,34,35,36]. Circulating Gal-9 could be involved in infection immunity by connecting the receptors of immune cells and various pathogens [37]. Studies reported the importance of soluble Gal-9 in acute viral infections, such as HIV [38,39,40], dengue virus [41], or influenza [42]. Moreover, as a potent intracellular modulator, Gal-9 is involved in a broad spectrum of cellular processes such as differentiation, proliferation, signaling, adhesion, migration, and apoptosis [43].

Recently, Gal-9 was defined as an immune-checkpoint molecule together with its receptor, T-cell immunoglobulin and mucin domain 3 (TIM-3) [44]. Gal-9 could bind to other cell-surface molecules such as CD44 [45], CD137 [46], and protein disulfide isomerase (PDI) [47] but there is mounting evidence that the interaction of Gal-9 with TIM-3 has a major role in the regulation of both innate and adaptive immunity. The presence of TIM-3 was proven in various innate and adaptive immune cells, including NK cells, T cells, DC, and macrophages [48,49]. The connection between Gal-9 and TIM-3 plays a crucial role in the apoptosis induction of Th1 and Th17 cells [50] and induces peripheral tolerance by modulating Treg differentiation and the Th1/Th2 balance [51]. Our previous study revealed a correlation between plasma Gal-9 levels and gestational age and showed that Gal-9+ Treg, TIM-3 + cytotoxic T, and NK cells could play an important role in the maintenance of healthy pregnancy [52,53]. It was found that dysregulated TIM-3 expression can be associated with effector cell dysfunction. While low or absent TIM-3 expression has been linked to excessive inflammatory responses and the development of autoimmune disorders, its overexpression contributes to an exhausted phenotype and inhibited response in chronic viral infections and cancers [54,55]. Blockade of the TIM-3 receptor can revert the exhausted phenotype of NK cells and improve their function in tumors [56].

Beyond the fact that NK-92MI cell lines and their cytotoxic potential against different malignancies were extensively studied both in in vivo and in vitro assays, moreover, a growing number of recently published trials used genetically chimeric antigen receptor (CAR)-transfected NK-92MI cells in tumor immunotherapy [57,58]. Besides the intensive research on NK cell lines in tumor immunotherapy, limited information is available on their immune-checkpoint expression and about the impact of different checkpoint ligands on their effector functions. Therefore, to uncover the therapeutic potential of modulating Gal-9-related immunological pathways in NK cell-based therapy, we investigated the dose-dependent effect of soluble Gal-9 on the TIM-3 checkpoint receptor and NKG2D, CD69, FasL, and perforin expression of NK-92MI cells. Moreover, we examined how their cytotoxicity and cytokine production were altered after Gal-9 treatment and in the presence of different serum supplements.

Our recent study provides evidence that the Gal-9/TIM-3 pathway plays an important role in the regulation of NK cell function and provides new data about the modulatory role of Gal-9 in the cytotoxicity and cytokine production of NK-92MI cells in the presence of different serum supplements. We hope that our results will aid the development of novel NK-cell-based strategies that target Gal-9/TIM-3 checkpoint in tumors resistant to T-cell-based immunotherapy.

## 2. Materials and Methods

### 2.1. Cell Culturing and Recombinant Galectin-9 Treatments

NK-92MI cells were cultured at 37 °C with 5% CO_2_ in alpha minimum essential medium (αMEM, Gibco, Waltham, MA, USA) supplemented with 12.5% horse serum and 12.5% fetal calf serum (FCS) (Gibco), containing 0.2 mM inositol (Sigma, Le Chesne, France), 0.1 mM 2-β mercaptoethanol (Gibco), 0.02 mM folic acid (Sigma), 100 U/mL penicillin, and 100 µg/mL streptomycin. To evaluate the effect of different serum additives, we added 10% FCS or 10% human AB serum (ABS) (Gibco) to the serum-free culturing medium and incubated the cell for 24 h at 37 °C. Treatment with human E. coli-derived recombinant Galectin-9 (Gal-9, Cat No:20-45GA, R&D Systems, Minneapolis, MN, USA) was performed in 30 and 100 nM concentrations for 24 h at 37 °C. K562 cells were maintained in Roswell Park Memorial Institute 1640 medium (RPMI, Lonza, Verviers, Belgium) supplemented with 10% FCS, 100 U/mL penicillin, and 100 µg/mL streptomycin (Lonza).

### 2.2. Surface and Intracellular Staining for Flow Cytometric Measuring

For surface staining, a total of 105 NK-92MI cells were incubated for 30 min at room temperature (RT) with different fluorochrome-labeled monoclonal antibodies (Table 1). After washing with Dulbecco’s phosphate-buffered saline without calcium and magnesium (DPBS, Lonza), the cells were resuspended in 300 μL PBS containing 1% paraformaldehyde (PFA), and stored at 4 °C in complete darkness until flow cytometric measuring. For intracellular staining, the surface-labeled NK-92MI cells were washed with PBS and fixed in 4% PFA for 10 min at RT. Next, the cells were washed again and incubated with 1:10 diluted FACS Permeabilizing Solution (BD Biosciences, San Diego, CA, USA) for 10 min at RT and then washed twice with PBS. Next, the cells were incubated with PE-conjugated mouse anti-human perforin antibody for 30 min at RT. The samples were then washed with PBS, fixed with PBS containing 1% PFA and stored at 4 °C in complete darkness until flow cytometric analysis. According to the manufacturer’s protocol, flow cytometer settings were checked with Cytometer Setup and Tracking beads (BD Biosciences). Labeled cells were analyzed with a FACS Canto II flow cytometer (BD Biosciences) equipped with the FACS DIVA V6. software (BD Biosciences) for data acquisition. Data were analyzed with FCS Express V4 software (De Novo, Pasadena, CA, USA).

### 2.3. Cytotoxic Assay for NK Cell Activity

The cytotoxic activity of NK-92MI cells was determined by FACS analysis according to previously published protocols [59,60,61]. Briefly, the target human erythroleukemia K562 cell line (1 × 10^6^) was pre-stained with a green fluorescent membrane dye PKH67 (Sigma). Then, 1 × 10^5^ NK-92MI effector cells were added to the K562 target cells in the effector:target (E:T) ratios of 12.5:1, 25:1, and 50:1. The mixtures were centrifuged for 5 min at 1250 rpm to pellet the cells together, and the tubes were incubated for 4 h at 37 °C in 5% CO_2_, in 10% FCS or 10% ABS-containing medium. After incubation, cell mixtures were labeled with propidium iodide solution (PI, 50 μg/mL, Sigma, Le Chesne, France). Dead target cells were identified by PKH67 and PI double positivity (Supplementary Figure S3). To determine spontaneous cell death, target cells incubated without the effector cells were used as controls. The percentage of killed target cells was calculated by subtracting background (spontaneous cell death) signals from experimental samples. Cytotoxicity was calculated as the percentage of killed target cells in each effector-to-target ratio.

### 2.4. Cytometric Bead Array (CBA)

For the determination of secreted cytokine levels, 1 × 10^6^ NK-92MI cells were aliquoted in 1 mL of media supplemented with different serum supplements and treated with 30 or 100 nM Gal-9 for 24 h at 37 °C. NK-92MI cells without the lectin were used as controls. After incubation, the cells were pelleted at 1250 rpm for 5 min; the supernatants were collected, aliquoted, and stored at −80 °C until further use.

For quantitative measurement of secreted cytokine levels, a Human Th1/Th2/Th17 Cytokine Bead Array (CBA) kit (BD Biosciences, San Diego, CA, USA) was used, which allowed for the simultaneous detection of IL-2, IL-4, IL-6, IL-10, TNF-α, IFN-γ, and IL-17A levels in the same sample. First, aliquoted supernatants were thawed, and CBA analysis was performed according to the manufacturer’s protocol. Briefly, microbeads coated with different cytokine-specific capture antibodies were mixed with each other. Next, 50 µL of the capture bead mixture was added to 50 µL of thawed supernatant samples. Then, 50 µL of phycoerythrin (PE)-conjugated detection antibody was added to the sample-bead compounds, and this mixture was incubated for 3 h in darkness at RT. The samples were then washed with 1 mL of wash buffer at 1100 rpm for 5 min, and the bead-pellets were resuspended in 300 µL of wash buffer. Cytokine standards were serially diluted to facilitate the construction of calibration curves necessary for the determination of cytokine concentrations in the test samples. Flow cytometric analysis of cytokine standards and samples was performed on a BD FACS Canto II flow cytometer with BD FACS DIVA software V6, and the obtained data were analyzed with FCS Express V4 software (De Novo).

### 2.5. Statistics

Two-way ANOVA in the R software package was used to test how the different serum additives and the various concentrations of soluble Gal-9 influenced the expression of the investigated surface receptors and intracellular molecules. In cases when the tested variables did not meet the criteria of normality, log_e_ transformations were performed. Gal-9, TIM-3, CD69, NKG2D, FasL, perforin, IL-2, IL-6, and IL-10 were loge transformed. The need for transformation was based on graphical evaluation, according to Crawley [62]. For pair-wise comparisons, Tukey post hoc tests were conducted with the multicomp-package [63] to compare each serum supplement/concentration combination with each other.

## 3. Results

### 3.1. Impact of Different Serum Supplements and Gal-9 Treatment on TIM-3 Receptor Expression and Surface Gal-9 Labeling

**Figure 1:**
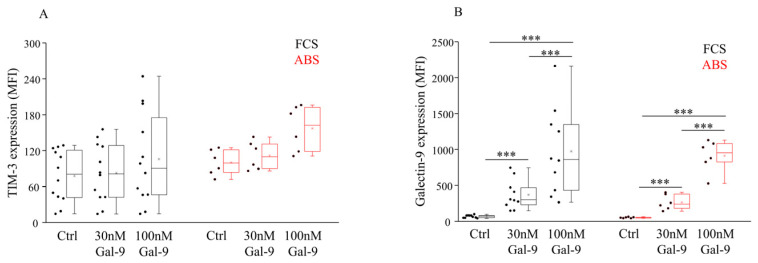
Surface receptor expression by NK-92MI cell line cultured in medium supplemented with FCS or human ABS and after recombinant Gal-9 treatment. The expression of TIM-3 (**A**) and Gal-9 (**B**) by NK-92MI cells following 30 and 100 nM Gal-9 addition, cultured in medium supplemented with 10% FCS or 10% human ABS. The solid bars represent medians of 10 determinations, the boxes indicate the interquartile ranges, and the lines show the most extreme observations. Differences were considered statistically significant for *p*-values *** < 0.01.

Investigating the TIM-3 receptor expression by NK-92MI cells cultured in the presence of FCS or ABS additive alone, a statistical difference was not observed in either case. Similarly, treatment with soluble Gal-9 had no statistically significant effect on the expression level of the TIM-3 receptor (Figure 1A, Supplementary Figure S1). However, it slightly increased the Mean Fluorescent Intensity (MFI) of TIM-3 + NK-92MI cells when human ABS was added to the medium. In comparison to the untreated control cells, incubation with 30 or 100 nM Gal-9 significantly (*p* < 0.01) and concentration-dependently increased the surface Gal-9 positivity by NK-92MI cells cultured in the presence of FCS or ABS (Figure 1B, Supplementary Figure S1).

### 3.2. Impact of Different Serum Supplements and Gal-9 Treatment on NKG2D and CD69 Receptor Expression

**Figure 2:**
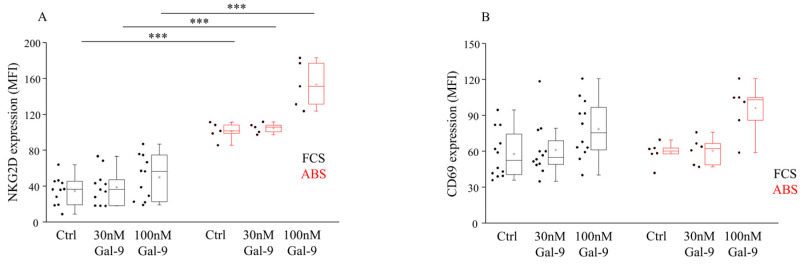
Activating receptor expressions by NK-92MI cell line cultured in medium supplemented with FCS or human ABS after recombinant Gal-9 treatment. The surface expression of activating NKG2D (**A**) and CD69 (**B**) receptors by NK-92MI cells following 30 and 100 nM Gal-9 addition cultured in medium supplemented with 10% FCS or 10% human ABS. The solid bars represent medians of 10 determinations, the boxes indicate the interquartile ranges, and the lines show the most extreme observations. Differences were considered statistically significant for *p*-values *** < 0.01.

Examining the expression of NK-activating receptor, significantly higher (*p* < 0.01) NKG2D MFI levels were detected when untreated cells were cultured in the presence of 10% ABS compared to FCS (Figure 2A, Supplementary Figure S1). No marked alteration was found in the expression of an early activation marker CD69 when different serum additives or an increasing concentration of Gal-9 was applied (Figure 2B). Moreover, 100 nM Gal-9 induced a slight elevation of both NKG2D and CD69 MFI values, but they did not reach statistical significance compared to the untreated cells, respectively.

### 3.3. Impact of Different Serum Supplements and Gal-9 Treatment on FasL Expression and Intracellular Perforin Content

**Figure 3:**
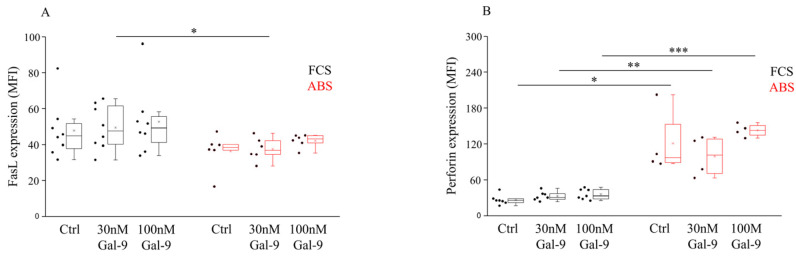
Surface FasL and intracellular perforin expression by NK-92MI cell line cultured in medium supplemented with FCS or human ABS and after recombinant Gal-9 treatment. The cell surface FasL (**A**) expression and intracellular perforin content (**B**) of NK-92MI cells following 30 and 100 nM Gal-9 addition, cultured in medium supplemented with 10% FCS or 10% human ABS. The solid bars represent medians of 10 determinations, the boxes indicate the interquartile ranges, and the lines show the most extreme observations. Differences were considered statistically significant for *p*-values * < 0.05; ** < 0.03; *** < 0.01.

In order to explore how serum supplements and extracellular Gal-9 could alter the death receptor-mediated apoptotic pathway and direct cytotoxic mechanisms of NK-92MI cells, the expression of the transmembrane ligand FasL and the intracellular level of perforin were analyzed with flow cytometry (Supplementary Figure S2.). We observed that soluble Gal-9 treatment had no remarkable effect on the cell-surface expression of FasL, except for treatment with 30 nM of Gal-9, which resulted in a significantly lower (*p* < 0.05) FasL MFI level in the presence of ABS (Figure 3A). Meanwhile, markedly higher (*p* < 0.05, *p* < 0.03) intracellular perforin expression was detected when NK-92MI cells were cultured with ABS compared to FCS (Figure 3B). No significant impact of the increasing concentration of Gal-9 on the intracellular perforin content was found (Figure 3B).

### 3.4. Effect of Different Serum Supplements and Gal-9 Treatment on the Cytotoxic Activity of NK-92MI Cell Line against K562 Target Cells

**Figure 4:**
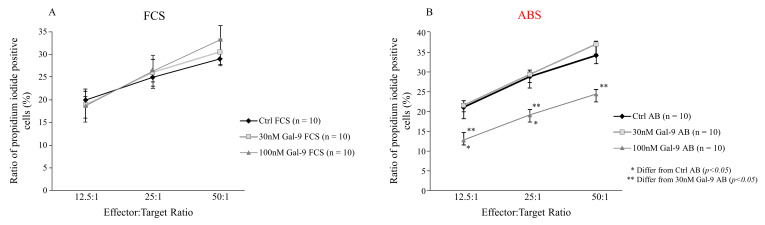
Cytotoxicity of NK-92MI cell line cultured in medium supplemented with FCS or human ABS and after recombinant Gal-9 treatment. Cytotoxic activity of NK-92MI cells following 30 and 100 nM Gal-9 addition, cultured in medium supplemented with 10% FCS (**A**) or 10% human ABS (**B**). Cytotoxicity is indicated as a percentage of lysed cells at different effector and target cell ratios. The results are expressed as the mean value ± standard error of the mean (SEM). Differences were considered statistically significant for *p*-values * < 0.05; ** < 0.03.

To uncover the functional impact of Gal-9 treatment on the direct cytotoxic activity of NK-92MI effector cells, a flow cytometry-based cytotoxicity assay was used under different culture conditions. In this assay, the human myelogenous leukemia cell line K562 was used as a target, and various effector:target (E:T) ratios were applied to reveal the dilution effect. After 4 h incubation, the functional test showed no significant or concentration-dependent alternations in the cytotoxic activity of NK-92MI cells cultured with 10% FCS (Figure 4A). Nonetheless, the ratio of dead (propidium-iodide positive) K562 cells was significantly reduced (*p* < 0.03) following 24 h addition of 100 nM Gal-9 and in the presence of 10% ABS. Pre-treatment with 10% ABS and 30 nM Gal-9 had no remarkable effect (Figure 4B) on the cytotoxicity towards K562.

### 3.5. Effect of Different Serum Supplements and Gal-9 Treatment on the Cytokine Production

**Figure 5:**
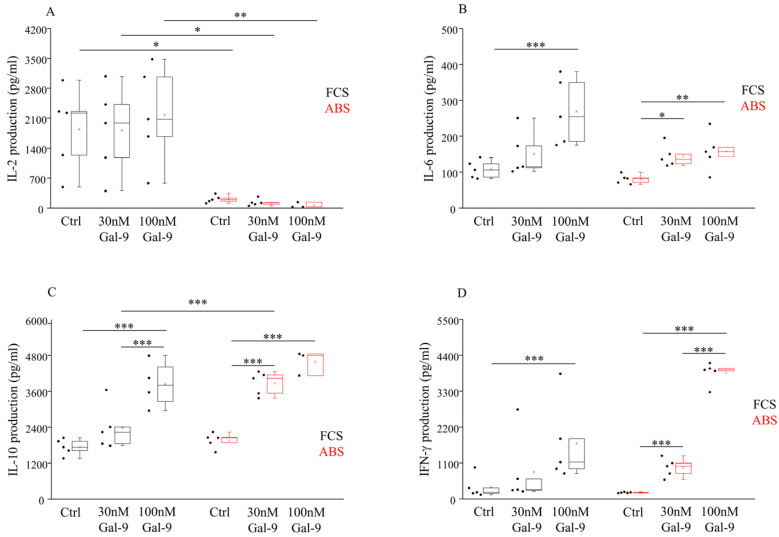
Different cytokine production by NK-92MI cell line cultured in medium supplemented with FCS or human ABS and after recombinant Gal-9 treatment. L-2 (**A**), IL-6 (**B**), IL-10 (**C**), and IFN-γ (**D**) cytokine production by NK-92MI cells, following addition of 30 and 100 nM Gal-9, cultured in medium supplemented with 10% FCS or 10% human ABS. The solid bars represent medians of 10 determinations, the boxes indicate the interquartile ranges, and the lines show the most extreme observations. Differences were considered statistically significant for *p*-values * < 0.05; ** < 0.03; *** < 0.01.

Finally, the influence of Gal-9 on the secretion of various Th1/Th2/Th17-type cytokines by IL-2 independent NK-92MI cells was tested by multiplex Cytometric Bead Array (CBA). A markedly higher (*p* < 0.05) IL-2 level was detected in the supernatant of NK-92MI cells cultured in an FCS-containing medium compared to ABS (Figure 5A). We could not detect any significant changes in the IL-2 production after treatment with soluble Gal-9. Compared to untreated cells, a significantly increased IL-6 production was found following 24 h treatment with 100 nM Gal-9, and the measured IL-6 levels were higher in the medium containing 10% FCS (*p* < 0.01) than in the medium with 10% ABS (*p* < 0.03) (Figure 5B). Furthermore, a moderately elevated (*p* < 0.05) IL-6 production was also found when the cell line was cultured with 30 nM Gal-9 and 10% ABS (Figure 5B). In the case of L-10 (Figure 5C), a significantly higher (*p* < 0.01) release was detected in the supernatant of NK-92MI cells after the addition of 30 or 100 nM Gal-9 in both culturing systems, suggesting a lectin dose-dependent IL-10 release compared to the untreated control cells. Additionally, a markedly elevated (*p* < 0.01) IL-10 content was also measured in the medium of 30 nM Gal-9-treated cells cultured with ABS compared to FCS. In comparison to the untreated cells, IFN-γ production by NK-92MI cells was significantly higher (*p* < 0.01) when cells were incubated with 10% FCS or ABS and after the addition of 100 nM Gal-9 (Figure 5D). Again, when cells were cultured with 10% ABS, their IFN-γ production was significantly elevated (*p* < 0.01) after 30 nM of Gal-9 addition (Figure 5D). The levels of IL-4, TNF-α, and IL-17 cytokines were below the detection limit of the CBA assay (data not shown).

## 4. Discussion

In our studies, we focused on Gal-9, which was originally described as an eosinophil chemoattractant and a potent immunomodulator [64]. While intracellular Gal-9 modulates cell proliferation, differentiation, apoptosis, and signal transduction [65], extracellular Gal-9 is involved in the control of adhesion, invasion, chemotaxis [43,66], and the regulation of local inflammation [67,68] and immunity [69]. It has anti-inflammatory properties and selectively induces apoptosis of Th1, Th17, and Type 1 CD8 + T cells. All of these effects are mainly mediated by the T-cell immunoglobulin and mucin domain 3 (TIM-3) receptor, which is the most extensively studied binding partner of Gal-9. Aside from TIM-3, Gal-9 can signal via other receptors on T-cells, so the outcome of Gal-9 signaling on T-cells likely depends on the specific receptor being activated by Gal-9, as well as the presence of additional (T-cell) skewing stimuli. Gooden MJ et al. implied that Gal-9 could have a Janus-like dual activity based on the cell type affected; it can inhibit immunity in autoimmune disease on the one side, and stimulate immunity in cancer and allergy on the other side [70]. Together, this suggests that the outcome of Gal-9 signaling varies greatly, depending on the cell type, the experimental conditions, and/or the balance of immunity in specific disease settings.

Previous data indicate that Gal-9 has a bidirectional role in malignant disorders that is influenced by the local concentration of Gal-9, the balance of immunity, and the micro-environmental circumstances or skewing signals related to various malignant conditions [23]. The biological role of Gal-9 depends on the histological type of tumors and the interactions between Gal-9 and its ligands expressed by malignant cells and immune effectors. Although a high intracellular Gal-9 level was found in various hematological malignancies and solid tumors, downregulated Gal-9 expression was reported in the progressive stage of melanoma [71], in hepatocellular carcinoma (HCC) [72], in breast cancer [73,74], and in cervix [75], gastric [76], and colon tumors [77]. Numerous earlier studies demonstrated that Gal-9 is essential for driving cancer formation by promoting the local expansion of Treg and myeloid-derived suppressor cells and enhancing the production of suppressive factors or cytokines [45,78,79]. Additionally, it was found that the release of Gal-9-containing exosomes from malignant cells induces apoptosis of TIM-3 + Th1 lymphocytes and aids the escape of tumors from immune recognition [80]. As recent evidence implicated that Gal-9 is one of the key TME factors that render tumors resistant to immunotherapy, it is reasonably plausible that blockage of the Gal-9-related pathway could be a promising therapeutic approach in certain malignancies.

In the context of targeting Gal-9 for cancer immunotherapy, it is crucial to carefully characterize the modulatory effects of Gal-9 on effector cells involved in the fight against tumors. NK cells could offer some significant advantages over T- or CAR-T-cell-based immunotherapy; hence, the primary aim of our study was to reveal the influence of soluble Gal-9 on the phenotype and function of NK cells. In our study, we chose the IL-2 independent NK-92MI cell line, as these “off-the-shelf” NK cells are free of contaminating T or B cells, thus mitigating any alloreactive or GvHD effects that may be associated with blood-derived primary NK cells. Furthermore, they can be safely cultured in vitro and expanded at a large scale, providing enough cells for research purposes and clinical applications. A further advantage of NK-92MI over other NK-derived cell lines is that they do not rely on IL-2, which makes them more suitable for clinical application. Additionally, the absence of most of the KIRs in NK-92MI results in a stronger cytotoxic response to malignant cells than primary NK cells; moreover, they can be easily modified with CARs, which exhibit a stronger anti-tumor effect both in vitro and in vivo, making them an ideal subject for adoptive therapy of CAR-T-resistant tumors.

In order to reveal the immunomodulatory effect of extracellular Gal-9 on NK-92MI cells, an increasing concentration of E. coli-derived human recombinant Gal-9 was added to the cells and the lectin-induced alterations of their phenotype, activating receptor expression, and biological function was examined under various cultivation conditions. Although fetal calf serum (FCS) is the standard culturing supplement in in vitro cell-based assays, besides FCS we performed parallel experiments with human AB serum (ABS) to uncover the influence of different culture conditions and for the better approximation of human physiological conditions.

First, we performed a multicolor flow cytometry-based phenotyping assay and observed that the treatment with recombinant Gal-9 significantly and concentration-dependently increased the surface Gal-9 labeling on NK-92MI cells, confirming that the lectin was successfully engaged by the receptors or binding partners expressed by these cells. The addition of different serum supplements did not alter the overall pattern of Gal-9 labeling under both conditions. Although neither 30 nor 100 nM Gal-9 could induce statistically significant changes in the TIM-3 receptor expression, a moderate increase in its MFI values was observed when 100 nM Gal-9 and 10% ABS were added to the cells. This effect was slightly masked by 10% FCS-containing medium. As the TIM-3 receptor is the main binding partner of extracellular Gal-9 [44], it is plausible that the increased TIM-3 expression that we measured following 24 h of 100 nM Gal-9 treatment could also be involved in the increased surface Gal-9 labeling of NK-92MI.

Besides these surface molecules, the expression of various activating NK receptors was also examined. We found that although 24 h treatment with 100 nM Gal-9 induced a moderate elevation of the activating NKG2D and CD69 receptor levels, they did not reach statistical significance. Interestingly, we noticed that the MFI levels of the most potent activating receptor, NKG2D, were strongly decreased in the presence of 10% FCS, which is in conjunction with the earlier results of Wiersma et al., who observed that the biological effects of Gal-9 could be masked by a yet unidentified component of FCS, but were unmasked when human plasma was used as a cell culture supplement. They found that the neutralizing effect of FCS is not merely due to binding of a serum component to Gal-9, but possibly, a serum component may shield the receptor(s) of Gal-9 on the tested cell lines [81]. Later, Aanhane et al. observed a similar neutralizing effect on the function of Gal-9 under high serum conditions [82] and reported that these masking components not only affected the ability of Gal-9 to hamper apoptosis but also altered its activity in other cell-specific functions, such as cell migration. Since it was found that the CD69 molecule is an early activation marker expressed on NK cells upon in vitro stimulus with IL-2 [83,84], and the NK-92MI cells transfected with the human IL-2 cDNA can effectively produce IL-2, it was not surprising that we could detect an elevated level of CD69 molecules on this cell line. Coincidently with the observed phenotypic changes after 100 mM lectin treatment, we suggest that the increased expression of the NKG2D, CD69, and TIM-3 receptors may mark Gal-9-mediated activation of these cells [85].

NK cells can kill target cells by using three major pathways [86]. While direct cytotoxicity is characterized by the release of perforin, granzyme, or granulysin from intracellular lytic granules of NK cells [87], death ligands can engage their cognate death receptors on the surface of target cells and induce apoptosis in a TNF-α-, TRAIL-, or FasL-dependent way. At last, IFN-γ plays a pleiotropic role in the stimulation of cellular immunity and possesses pro-apoptotic, anti-proliferative, and cytostatic effects against tumor cells. Moreover, IFN-γ inhibits angiogenesis, induces apoptosis of Treg cells, and stimulates pro-inflammatory M1 macrophages to overcome tumor progression [88].

In our study, neither the serum additives nor the Gal-9 treatment could induce statistically significant changes in the FasL expression of NK-92MI cells. However, a significantly higher basal perforin level was detected when human AB serum was added to the medium. Although a slightly increased perforin expression was induced following 100 nM Gal-9 treatment, a statistically significant difference was not found between the untreated or lectin-treated cells. Here again, a strong masking effect of FCS was discovered. In contrast with our results, Golden-Mason and colleagues previously found that perforin mRNA expression was significantly downregulated in NK cells following the addition of Gal-9 [89], although their test conditions were remarkably different from ours. First, they used the IL-2- dependent NK-92 cell line instead of IL-2 independent NK-92MI cells. Second, they treated the cells only in FCS-containing medium but not in the presence of ABS. Next, they used a higher concentration of Gal-9 for treatment (5 ug/mL, 150 nM), contrary to the lower levels (30, 100 nM) that we applied. Finally, they treated the NK cells with recombinant Gal-9 molecule provided by GalPharma, but we used R&D-produced lectin.

To uncover the impact of Gal-9 treatment on the killing potential of NK-92MI cells, a flow cytometry-based cytotoxic assay was applied. After pre-incubating the effector cells for 24 h with 100 mM of Gal-9 and 10% ABS, a significantly decreased K562-killing was observed at all E:T ratios. In the presence of 10% FCS, none of the used lectin concentrations affected the cytotoxicity, providing again strong support of the FCS-related blocking effect on the biological activity of Gal-9. Though Gal-9 caused a moderate increase in the MFI of intracellular perforin levels, we suppose that the discordance in the elevated perforin content and the decreased cytotoxicity against K562 cells might have resulted from (1) the inhibited degranulation (e.g., decreased CD107a expression), (2) dysregulated lytic pathways (e.g., decreased granzyme or death-ligand expression), or (3) by other indirect inhibitor mechanisms (e.g., increased inhibitory cytokine secretion) that might be involved in the regulation of NK-cell-mediated cytotoxicity. These observations were in conjunction with the previously published results of Motamedi et al., who reported that the expression of TIM-3 receptor on CD56+/CD16+ NK cells correlated with higher intracellular granzyme B and perforin expression. Similar to our results, they found that the Gal-9 + NK subpopulations of HIV-infected patients exhibited impaired cytotoxic activity but showed increased IFN-γ production ability compared with their Gal-9-negative counterparts [39]. However, the exact underlying mechanisms explaining this paradox have yet to be established.

Because the NK-92MI cell line used in this study contains, expresses, and synthesizes IL-2 [15], the high level of IL-2 that we detected in the FCS-containing supernatant of untreated NK-92MI cells was reasonable. Surprisingly, the supernatant of the 10% ABS-supplemented cells contained a significantly lower amount of IL-2 (FCS vs. ABS (mean ± SD) = 1840.03 ± 977.7 vs. 207.7 ± 85.95 pg/mL). Based on the observations that the ABS-supplemented NK-92MI cells proliferate similarly to the FCS-treated ones and we could not detect significant differences in their spontaneous cell death (FCS vs. ABS (mean ± SD) = 6.91 ± 4.95 vs. 4.74 ± 1.29), we can exclude that this phenomenon originated from the decreased viability of the ABS-supplemented cells. In addition, as the performance of the Cytometric Bead Array for the determination of cytokine levels in the supernatant of NK-92MI cells was optimized for analysis of tissue culture supernatants, EDTA plasma, and serum samples, the presence of human AB serum in the examined culture media should not cause any detection problems. Therefore, one possible explanation might be based on the paper of Gong et al. [12], who reported that the expression of the CD25 (IL-2Rα) molecule on the surface of NK-92 cells correlated inversely with the IL-2 level of the culturing media. Since the level of endogenously produced IL-2 by the NK-92MI cell line is high, downregulation of the CD25 receptor is logically expected, as it was reported earlier by Tam et al. [15]. It seems feasible that under certain activating circumstances (e.g., in the presence of human AB serum and/or Gal-9), the expression of the CD25 molecule or other IL-2R subunits can be upregulated. It is probable that the increased number of IL-2R on the surface of NK-92MI cells can anchor secreted IL-2 to the plasma membrane, resulting in subsequent consumption of the detectable cytokine from the culture medium [90]. Because it is known that the parental NK-92 cell line expresses all of the IL-2R subunits (CD25, CD122, CD132), we can imagine that these molecules can be involved in the binding of secreted IL-2 on the surface of NK-92MI cells. However, in the absence of prior supporting data, further studies are needed to confirm this hypothesis.

NK cells are an early and major innate source of IFN-γ [91], which either directly inhibits carcinogenesis or facilitates the immune recognition of tumors [92] and promotes adaptive immunity [91,93]. In our present study, we verified that the NK-92MI cell line expresses the TIM-3 immune checkpoint receptor, which is slightly and dose-dependently upregulated after 24 h treatment with soluble Gal-9. In agreement with the previously published results of Gleason et al. [94], we affirmed that at all concentrations tested, Gal-9 was able to induce significantly higher IFN-γ production in a dose-dependent fashion when 10% ABS was used as a culture supplement. We have to note that while they reported that >50 nM of Gal-9 could induce apoptosis in NK-92 cells, we did not observe an increased number of dead cells 24 h following 100 nM Gal-9 treatment. The addition of 10% FCS strongly masked this effect. However, our present data strongly support that engagement of TIM-3 by Gal-9 on NK-92MI cells is specific, and the activation of the TIM-3/Gal-9 pathway is markedly involved in the elevated IFN-γ production of these cells. We propose that treatment with soluble Gal-9 skewed TIM-3 + NK-92MI cells toward cytokine-producing effectors, rather than enhancing direct cytotoxicity or inducing NK-cell exhaustion or dysfunction [39].

As NK-92MI cells have a CD56 ^bright^-like phenotype [95], besides IFN-γ, they can produce various cytokines and chemokines, including GM-CSF, IL-10, IL-5, IL-13, RANTES, TNF-α, MIP-1α, MIP-1β, and IL-8 [4]. In our study, 24 h treatment with soluble Gal-9 induced a significant and dose-dependent increase in IL-6 and IL-10 production under both FCS- and ABS-laden culturing conditions. It was found that those disorders that are characterized by persistently elevated levels of IL-6 exhibit diminished NK-cell function, including endometriosis, systemic juvenile idiopathic arthritis, heart failure, HIV, and severe COVID-19 [96,97]. In vitro studies revealed that IL-6 significantly reduces the cytotoxicity of human peripheral NK cells and downregulates the expression of intracellular perforin, granzyme, and the early degranulation marker CD107a [98]. In addition, IL-6 induces anergy to other cytokines by long-term induction of suppressor of cytokine signaling (SOCS) protein [99]. Based on these literature data, we suppose that the significantly increased IL-6 production following 100 nM Gal-9 and 10% ABS treatment at least partly explains the diminished lytic activity of NK-92MI cells against K562 target cells.

Moreover, we found that Gal-9 treatment triggered significantly higher IL-10 production by NK-92MI cells in a dose-dependent fashion and observed that this stimulating effect was more prominent in the presence of 10% ABS. It is well accepted now that IL-10 is a potent immunosuppressive cytokine that promotes escape of tumor cells from immunosurveillance mediated by antigen-presenting cells and T-helper cells, and, as a consequence, decreases the cytotoxic function of NK cells mainly in a secondary manner [100]. On the other hand, new scientific evidence supports that IL-10 exerts a stimulatory effect on NK cells and enhances their proliferation, cytotoxicity, and IFN-γ production, and when combined with IL-15 or IL-18 [101,102]. Indeed, further research is required to define the exact functional consequences of the increased IL-10 and IL-6 production after Gal-9 treatment and the role of the TIM-3/Gal-9 signaling pathway in their biological function.

## 5. Conclusions

**Figure 6:**
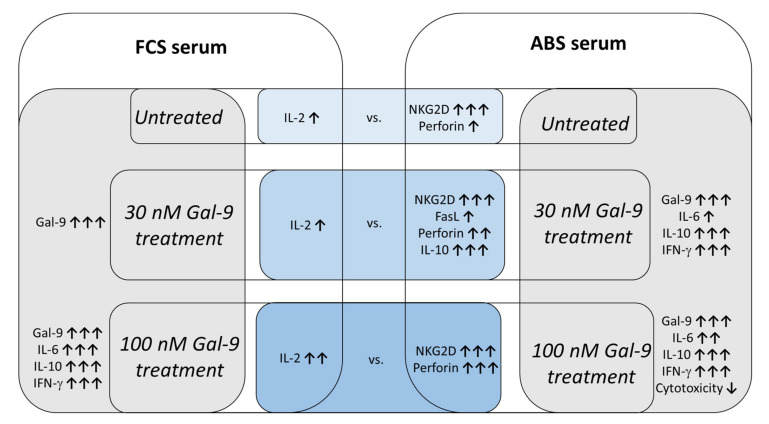
Comparing the effects of the two different serum supplements following recombinant Galectin-9 treatment. Upwards pointing arrows indicate an increase in a numerical value, and downwards pointing arrows indicate a decrease.

Collectively, our data suggest that the expression of the TIM-3 immune checkpoint receptor can be induced on NK-92MI cells by recombinant Gal-9 treatment, and the elevated level of TIM-3-, CD69-, and NKG2D-activating receptors may mark a dose-dependent activation of these cells, which is strongly masked in the presence of FCS-containing media, but is unmasked when 10% ABS is applied as a culture supplement (Figure 6). Although we found that Gal-9 treatment significantly increased the production of IFN-γ, IL-6, and IL-10 in a dose-dependent manner, the higher lectin level markedly inhibited the in vitro cytotoxic activity of the ABS-cultivated effector NK cells against the target K562 cell line. Since the intracellular perforin content of NK-92MI cells was slightly but not significantly elevated after lectin treatment, we assume that the dysregulated or disconnected lytic pathways or the increased deliberation of inhibitory cytokines might be involved in the Gal-9-dependent local suppression of cytotoxicity of NK-92MI cells (Figure 6). The increased deliberation of IFN-γ from NK-92MI cells following TIM-3/Gal-9 ligation could activate cytotoxic lymphoid cells to foster the attack of malignant cells that break off from the primary tumor, preventing their circulation and the formation of metastasis.

Finally, with respect to our recent results, the use of human pooled AB serum instead of FCS appears prudent for in vitro assays on NK-derived cell lines, in particular when examining the biological and regulatory role of Gal-9 or other members of the galectin family, to avoid the strong masking effect of FCS on their activity.

We hope that our recent results may aid the development of novel NK-cell-line-based strategies that target immune checkpoints against tumors resistant to T-cell-based immunotherapy; however, we must note that the present study has limitations. First, the tested sample size and the available reagents that we used for phenotypic and functional characterization of NK-92MI cell lines were quite limited. Next, the blocking experiment was not fulfilled in this research to verify the role of the TIM-3 immune checkpoint in the observed alterations after Gal-9 treatment. As a consequence, only hypotheses and theoretical conclusions could be made, although prior studies or previously published results support our suggestions. Nonetheless, the open questions and the probable underlying mechanisms remain unsolved and need to be explained in the future.

## Figures and Tables

**Table 1:** Fluorochrome-conjugated monoclonal antibodies used in the study.

Antigen	Format	Clone	Isotype	Company	CAT
CD69	BV421	UCHT1	Mouse BALB/c IgG1,κ	BD Biosciences	563109
Galectin-9	PE	9M1-3	Mouse IgG1, κ	Biolegend	348906
FasL	PE	NOK-1	Mouse IgG1, κ	Biolegend	306407
NKG2D	PE-Cy7	1D11	Mouse RBF/DnJ IgG1, κ	BD Biosciences	562365
Perforin	PE	δG9	Mouse BALB/c IgG2b, κ	BD Biosciences	556437
TIM-3	APC	344823	Rat IgG2A	R&D Systems	FAB2365A

## Data Availability

The data presented in this study are available on request from the corresponding author. The data are not publicly available.

## Supplementary Materials

The following are available online at https://www.mdpi.com/article/10.3390/biom11081066/s1, Figure S1: The expression of TIM-3, Gal-9 and NKG2D by NK-92MI cells, Figure S2: The expression of CD69, FasL and Perforin by NK-92MI cells, Figure S3: The expression of the PKH molecule by the K562 cells and determining the frequency of propidium iodide positive cells.
