# Supplementary material for: Influence of Galectin-9 Treatment on the Phenotype and Function of NK-92MI Cells in the Presence of Different Serum Supplements
Source: Biomolecules. 2021 Jul 22;11(8):1066. doi: 10.3390/biom11081066 (PMC8391477; doi:10.3390/biom11081066)
Supplement: Supplementary file 1 [file biomolecules-11-01066-s001.zip › biomolecules-1287733-supplementary.pdf]

Supplementary Figure S1.

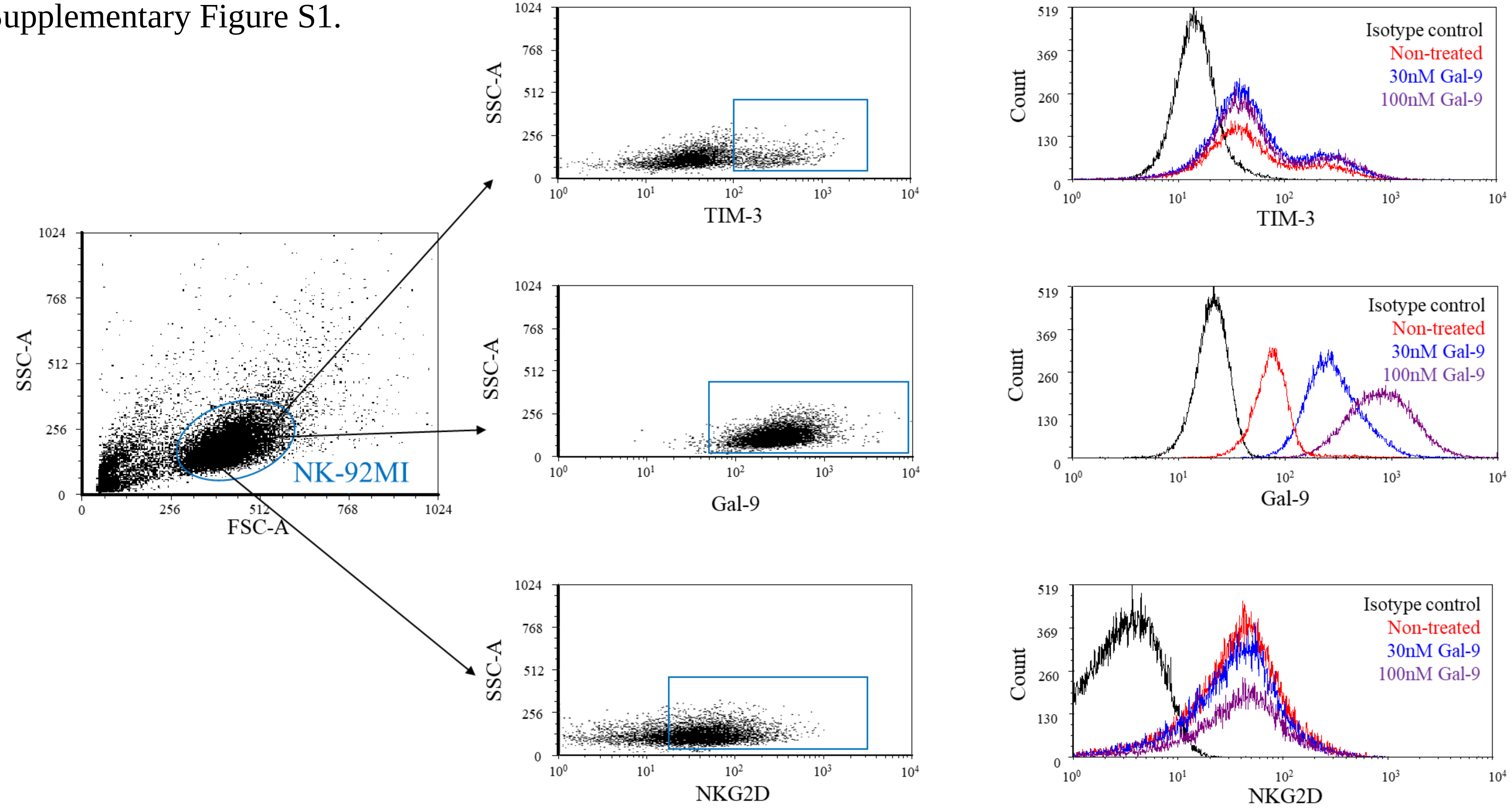

Supplementary Figure S1: The expression of TIM-3, Gal-9 and NKG2D by NK-92MI cells.

Representative FACS plots and histograms show the surface expression of TIM-3, Gal-9, and NKG2D by NK-92MI cells. Based on the FSC-A/SSC-A parameters NK-92MI cells were gated and the positivity of the surface molecules (TIM-3, Gal-9 and NKG2D) was determined compared to the isotype controls.

Supplementary Figure S2.

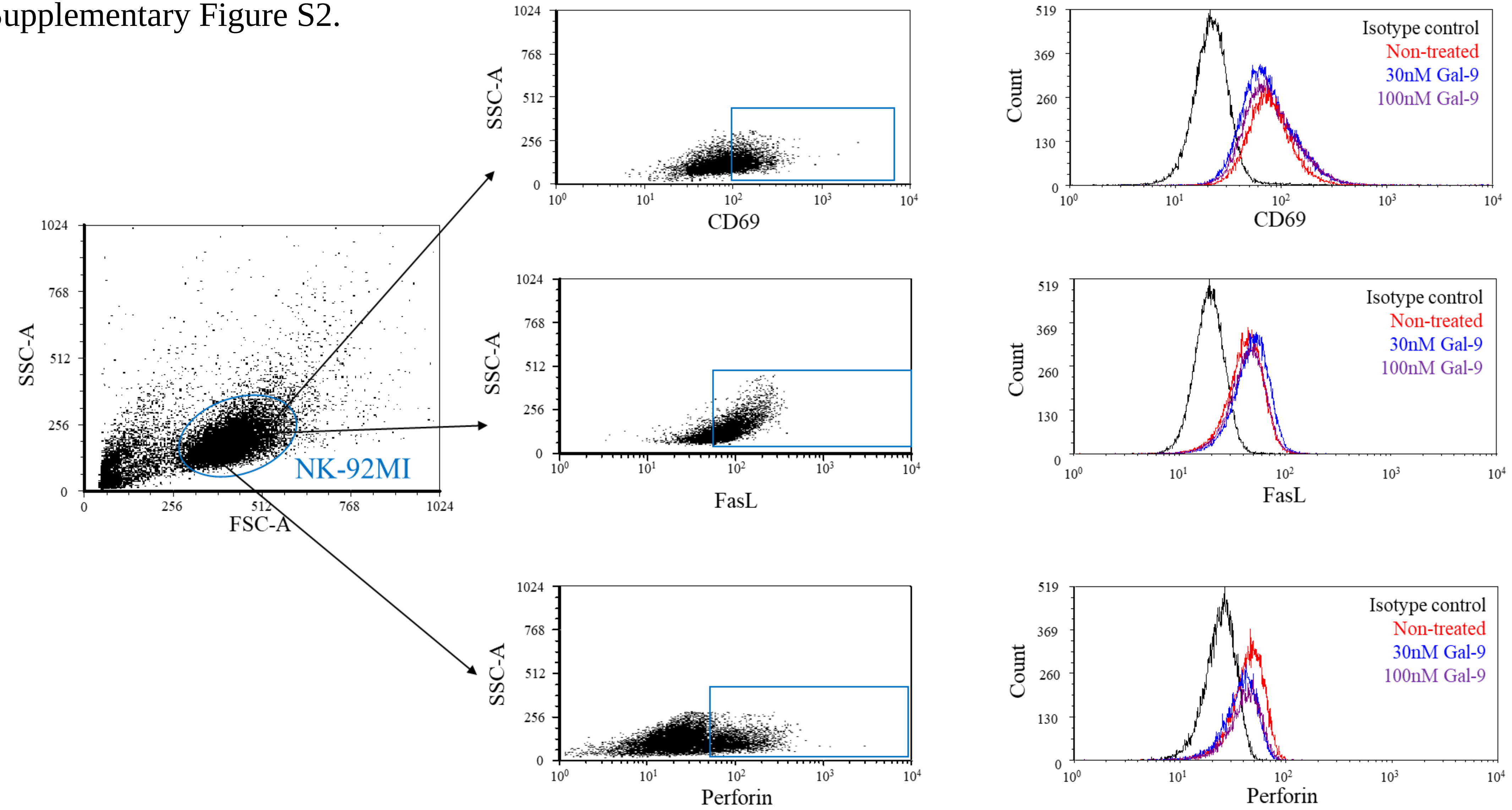

Supplementary Figure S2: The expression of CD69, FasL and Perforin by NK-92MI cells.

Representative FACS plots and histograms show the surface expression of CD69, FasL, and the intracellular expression of perforin by NK-92MI cells. Based on the FSC-A/SSC-A parameters NK-92MI cells were gated and the positivity of the surface molecules (CD69, FasL) and the intracellular perforin was determined compared to the isotype controls.

Supplementary Figure S3.

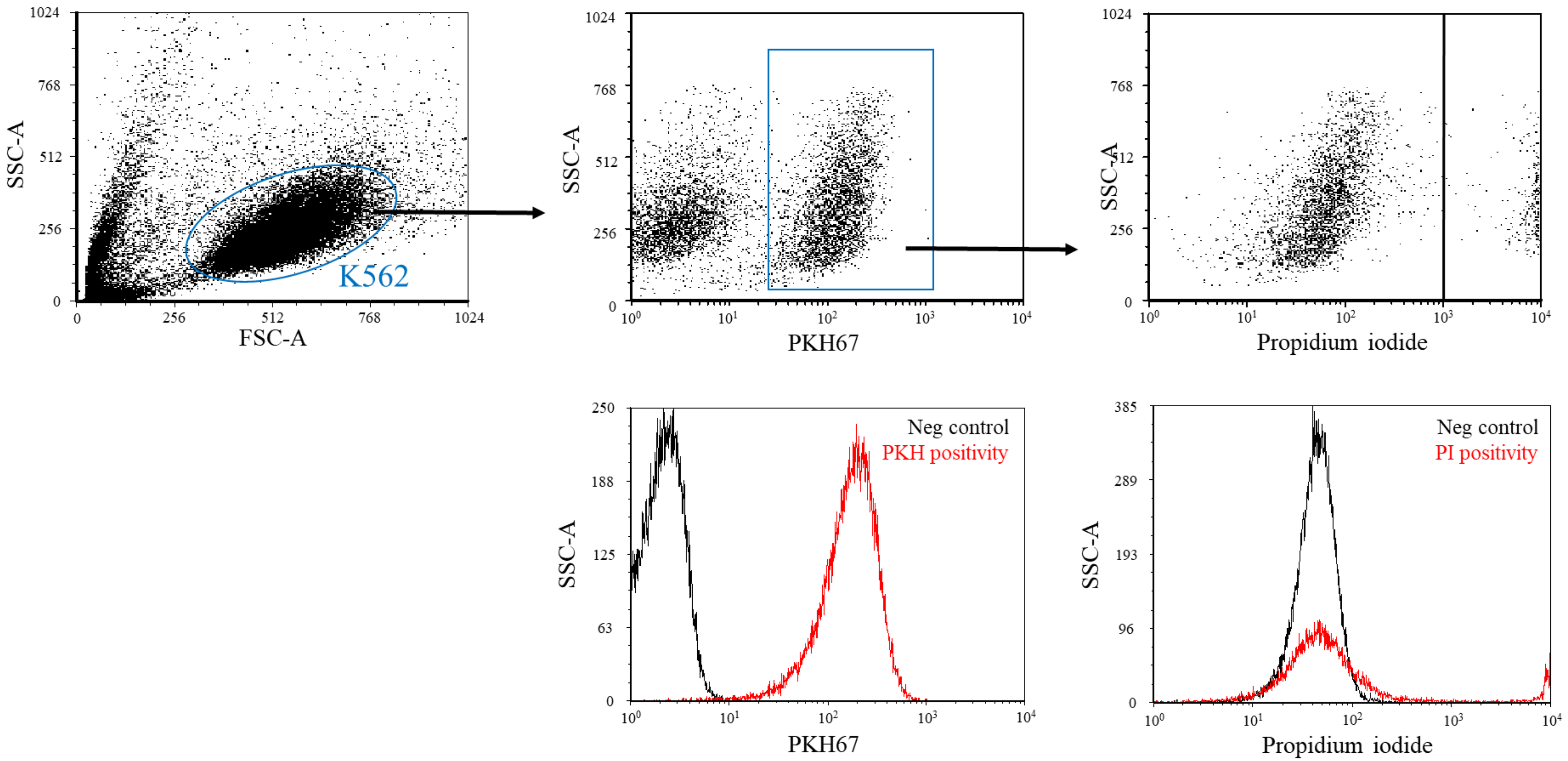

Supplementary Figure S3: The expression of the PKH molecule by the K562 cells and determining the frequency of propidium iodide positive cells.

Cytotoxic activity of NK-92MI cells was evaluated by FACS analysis. Based on the FSC-A/SSC-A parameters K562 cells were gated. Dead target cells were identified by simultaneously expressed PKH67 and propidium iodide molecules.
